# A quality assessment of clinical practice guidelines with recommendations for family involvement in the care of individuals diagnosed with schizophrenia, bipolar mood disorder, and major depressive disorder: Critical appraisal utilizing AGREE II

**DOI:** 10.3389/fpsyt.2022.1065129

**Published:** 2023-01-04

**Authors:** Raziye Dehbozorgi, Malek Fereidooni-Moghadam, Mohsen Shahriari, Ebrahim Moghimi-Sarani

**Affiliations:** ^1^School of Nursing and Midwifery, Isfahan University of Medical Sciences, Isfahan, Iran; ^2^Community Based Psychiatric Care Research Center, School of Nursing and Midwifery, Shiraz University of Medical Sciences, Shiraz, Iran; ^3^Research Center for Psychiatry and Behavior Science, Shiraz University of Medical Sciences, Shiraz, Iran

**Keywords:** practice guideline, family, schizophrenia, bipolar mood disorder, major depressive disorder

## Abstract

**Introduction:**

Evidence suggests that family-center collaborative care is useful for individuals identified with chronic mental illness. Clinical practice guidelines offer specific recommendations primarily based on to be had studies and are beneficial in informing evidence-based practice and guiding destiny studies.

**Objective:**

Identify current scientific practice guidelines including family-center collaborative care suggestions for individuals with Bipolar Mood Disorder, Schizophrenia, and Major Depressive Disorder and analyze the selection of guidelines for their methodological quality.

**Methods:**

A systematic search was conducted on seven electronic databases (G-I-N), (NICE), (MOH), (SIGN), (WHO), (NIH) and (APA) and additional sources. Three referees independently reviewed articles and selected guidelines for inclusion criteria. Subsequently, 18 trained appraisers independently assessed all 15 guidelines using AGREE II.

**Results:**

The mean scores for domains and overall quality were computed. For the overall assessment of the guidelines, 60% reached the quality threshold with domain scores of 60%. The overall average quality rating for these guidelines was 58/29%.

**Conclusion:**

The applicability of the guidelines needs to be improved in order to improve their relevance and clinical utilization. As individuals with chronic mental illnesses progress through their disease course, families and health care providers play a crucial role in helping them. The analysis of research knowledge on effective rehabilitation techniques, including the involvement of families in treatment, can be enhanced by using well-developed and appropriate methods.

## Introduction

Schizophrenia, bipolar mood disorder (BMD) and major depressive disorder (MDD) are serious and persistent mental disorders that cause significant impairments and place a heavy burden on caregivers and families (1–5). To provide evidence-based mental health care, caregivers must be skilled, have evidence-based knowledge, monitor patients continuously, and take a holistic approach and despite recent global commitments, countries have performed poorly, inefficiently, and fragmentedly in managing chronic mental illnesses despite the many serious problems they face and the burden they impose on their families, economies, and societies (1, 6). In order to assist patients with special needs, family-centered collaborative care (FCCC) can benefit patients, families, healthcare providers (HCPs), and service providers (7, 8). A critical component of FCCC is the involvement of the family as an expert and partner in all care systems. Care and services must be enhanced in the health system to achieve positive patient and organizational outcomes. By providing performance support, reducing unnecessary interventions, increasing knowledge and skills, establishing leadership roles for HCPs, and enhancing competence, the quality of care can be improved by clinical practice guidelines (CPGs) (9, 10). CPGs can assist caregivers and HCPs in developing and implementing evidence-based interventions. As well as identifying gaps in the evidence base, these tools can guide future research. A systematic review of guidelines recommending FCCC was conducted by the research team despite evidence suggesting that CPGs improve clinical practice, questions remain regarding guideline development and quality (11–13). The best available evidence should be used to inform the development of published guidelines (13, 14). Concerns about the quality of CPGs may arise because guideline committees may be biased, which has an impact on the recommendations they make (15). In addition, recommendations on one topic from different organizations also vary (16, 17). As a result, guidelines need to be developed and submitted in a consistent and rigorous manner despite recommendations for the appropriate methodology (17–19), an earlier study assessing the quality of CPGs between 1985 and 1997 found that the guidelines did not meet established methodological standards (20). The development of guidelines is also of considerable importance with respect to conflict of interest reporting (21). CPGs play a significant role in health care, and making their development process more transparent would help to reduce conflicts of interest. As part of a recent agreement on guideline development, the council demanded that members of the guideline development group disclose conflicts of interest, but failed to include this information within the guideline (22).

It is important to ensure that the method of developing guidelines is free of biases, that recommendations are internally and externally valid, and that recommendations are clinically relevant (23). Increasing complexity, heterogeneity, and number of clinical guidelines has led to the need for internationally recognized standards for assessing clinical guidelines' quality (24, 25).

Despite the fact that CPGs are increasingly being used to treat patients with severe and persistent mental disorders, since their quality is unstable, there have been concerns regarding their reliability, which has implications for methodological evaluation, and can adversely affect the usefulness and benefits of guidelines (25–27). Conflict of interests are also important elements of AGREE II (Appraisal of guidelines for research and evaluation), a validated questionnaire used to evaluate CPG's methodological quality (22). A systematic review of additional assessment tools utilized to assess CPGs discovered AGREE had the most potential to function as guidelines critical appraisal tools (28). The use of the AGREE II instrument is nowadays general in published scientific analyses and it has also been adopted by major health bodies in their evaluation of CPGs (29).

Thus, this analysis aimed to evaluate the quality of CPGs for the FCCC of individuals with schizophrenia, BMD, and MDD published over the last 13 years by utilizing AGREE II instrument to clinically involve them with patients to improve evidence-based judgments and enhance the safety of the patients and the HCPs.

## Materials and methods

### Section 1: Systematic review of the literatures

#### Search strategy

**Figure 1:**
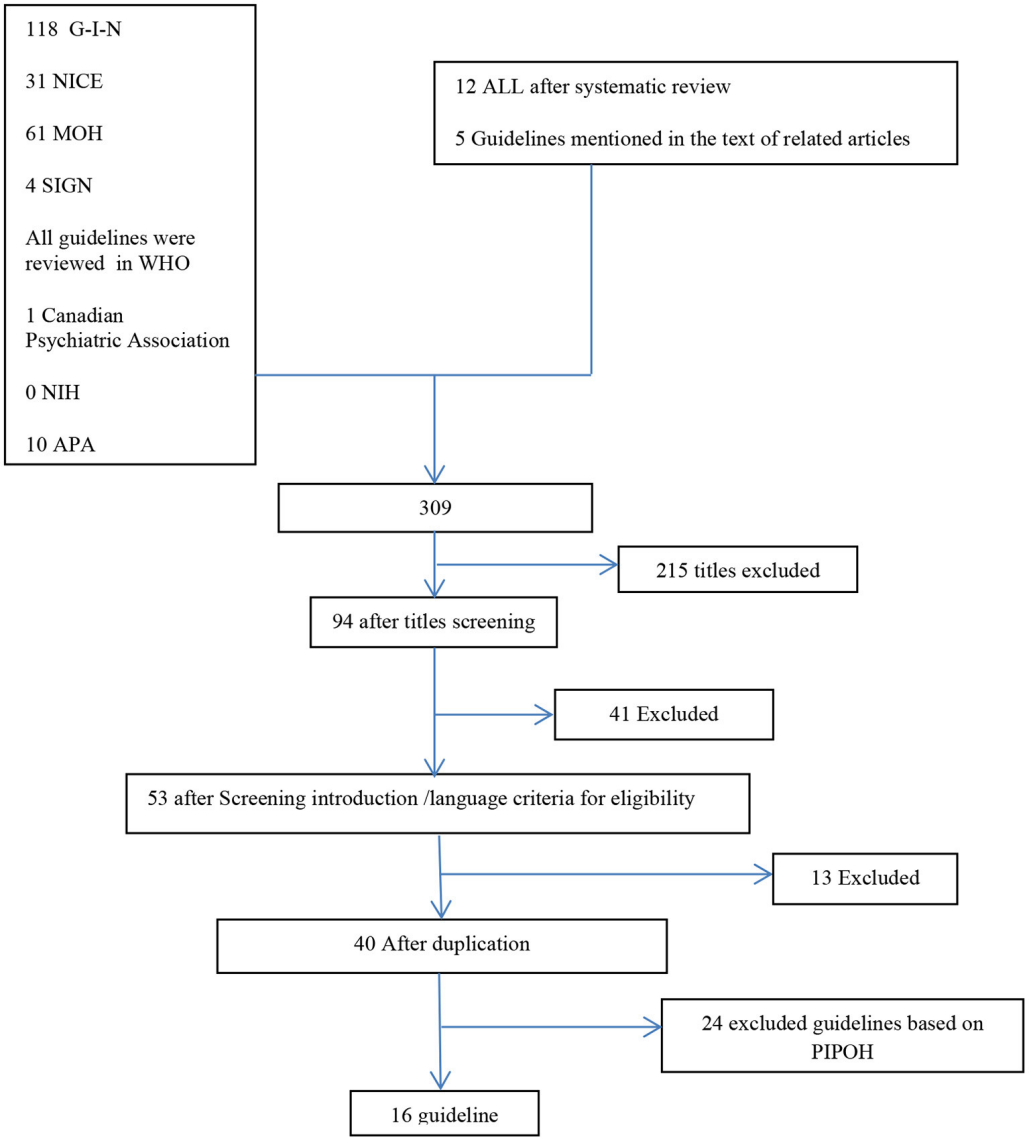
Diagram for the inclusion and exclusion of guidelines.

The literatures were systematically reviewed to determine current CPGs with FCCC recommendations for individuals diagnosed with schizophrenia, BMD, and MDD. Search strategies were designed by the researchers and checked by a librarian at the Isfahan University of Medical Sciences Library. We used the separate search terms according to each database's rules but in general we used “collaborative care^*^” AND “chronic mental disorder” OR “severe and persistent mental disorder” OR “Schizophrenia” OR “Bipolar mood disorder” OR “Major Depressive Disorder” AND “family-center care^*^”. The search strategies reported in Figure 1. In 2021/1/20, searches were conducted in the following electronic databases: (G-I-N: Guideline International Guideline), (NICE), (MOH: Ministry of Health), (SIGN: The Scottish Intercollegiate Guidelines Network), (WHO), (NIH: National Institutes of Health), and (APA: American Psychological Association). Subsequently, supplemental searches were conducted by hand searching reference lists of key and appropriate articles after systematic review as well as guidelines mentioned in the text of related articles, and the Canadian Psychiatric Association was searched.

#### Guideline selection

Using the EndNote X7 software, the primary reviewer (RD) provided first screening of titles to remove unessential guidelines. The remaining articles were reviewed at the abstract level, reading the introduction and application of English language entry criteria, and at the full-text level by two separate reviewers (MF and MSH). Relevant guidelines were selected according to the selection criteria, reported in Figure 1. English language CPGs which recently developed in the range of 2000–2022 years were included, and provided specific recommendations on at least one mental illness and FCCC for adults (aged 18 years and older) with schizophrenia, BMD, and MDD. In possibilities where guidelines were updated, guidelines were reviewed to obtain relevant points related to methodology. Differences in article selection were solved through consultation with a fourth reviewer (EMS) to achieve agreement. Table 1 showed the report of included guidelines with FCCC recommendations for individuals diagnosed with Schizophrenia, BMD, and MDD.

**Table 1:** Description of included guidelines with FCCC recommendations for individuals diagnosed with Schizophrenia, BMD, and MDD.

**Number**	**Guideline**	**Organization (country)** **(in English)**	**Title**	**Topic**
1	NICE (30)	England	Bipolar disorder in adults	Bipolar disorder
2	NICE (31)	England	Common mental health problems: identification and pathways to care Clinical guideline	Common mental health problems
3	NICE (32)	England	Supporting adult carers NICE guideline	adult carers
4	Crawford et al. (33)	England	Service user experience in adult mental health: improving the experience of care for people using adult NHS mental health services	Care of people using adult mental health services
5	NICE (34)	England	Depression in adults with a chronic physical health problem: recognition and management Clinical guideline	Depression
6	NICE (35)	England	Violence and aggression: short-term management in mental health, health and community settings NICE guideline Published: 28 May 2015	Violence and aggression
7	NICE (36)	England	Violent and aggressive behaviors in people with mental health problems	Violence and aggression in people with mental health problems
8	Thornicroft et al. (37)	Switzerland	Management of physical health conditions in adults with severe mental disorders	physical health conditions in adults with severe mental disorders
9	MOH (38)	Malaysia	Management of Bipolar Disorder in Adults	Bipolar Disorder
10	Santa Mina et al. (39)	Canada	Assessment and Care of Adults at Risk for Suicidal Ideation and Behavior	Suicidal Ideation and behavior
11	Couroupis et al. (40)	Australia	Australian Clinical Guidelines for Early Psychosis	Psychosis
12	NICE (41)	England	Coexisting severe mental illness and substance misuse: community health and social care services	severe mental illness and substance misuse
13	de la Cámara Izquierdo et al. (42)	Spain	Clinical Practice Guidelines for Psychosocial Interventions in Severe Mental Illness	Psychosocial Interventions in Severe Mental Illness
14	Addington et al. (43)	Canada	Canadian Practice Guidelines for Comprehensive Community Treatment for Schizophrenia and Schizophrenia Spectrum Disorders	Schizophrenia
15	Keepers et al. (44)	USA	The American psychiatric association practice guideline for the treatment of patients with schizophrenia third edition	Schizophrenia

### Section 2: Guidelines critical appraisal

#### The instrument

To assess the methodological quality of guidelines, AGREE II tool has been validated and is a trustworthy instrument. Each item in the questionnaire is scored on a seven-point scale, and they categorized into six domains: A. scope and purpose; B. stakeholder involvement; C. rigorous development; D. clarity of presentation; E. applicability; and F. editorial independence (22, 45).

#### Training of appraisers

Twenty three appraisers initially agreed to evaluate 16 clinical guidelines, 5 appraisers withdrew, and 18 appraisers agreed to complete the evaluation. The inclusion criteria of the appraisers were the experience of working in the field of psychiatric diseases for at least 3 years and willingness to cooperate in each stage of the evaluation. Even if they showed unwillingness in the middle of the evaluation, they were excluded from the study. Eighteen appraisers were trained on the AGREE II instrument with a reviewer (RD) and the first assessment was done in the presence of the trainer to ensure the accuracy of the evaluation (22). AGREE II was used by the appraisers before this review to familiarize themselves with the instrument.

#### Appraisal of guidelines

Included clinical guidelines were presented to the relevant professionals for evaluation with AGREE II. This tool was used to review the clinical guidelines according to the publication year, the organization that developed it, the quality of evidence, and the target population. To appraise clinical guidelines, the researcher contacted the health system staff presently or through phone. After that, carrying an invitation note for collaboration including a short description of the methodology and goals of the study, she saw their offices or workplaces and after obtaining informed consent from the appraisers, they were provided with a CD including 1–4 clinical practice guidelines with the AGREE II tool, its completion guide and essential explanations on how to assess the guidelines, and all of their questions were answered. According to the advice in AGREE II manual, the individual guideline is assessed by at least 3–5 appraisers, as raising the number of appraisers will improve the trustworthiness of the appraisal (22). Therefore, in this study, each guideline was appraised by 18 HCPs from different disciplinary consisting of a psychiatric, psychologist, and mental health nurse to enhance the reliability of the appraisal who were not members of the research team. From 2021/7/5 to 2021/10/16, clinical guidelines were reviewed and appraised. A total of 18 appraisers independently assessed each guideline included in the review using the AGREE II instrument with the user's manual (22). Two to four appraisers evaluated each guideline to ensure validity. A 7-point scale was used to grade the 23 AGREE II items, with 1 meaning that a single item did not meet any of its criteria or was poorly reported, and 7 meaning that each item satisfied all its criteria and was well reported. A quality assessment score was also assigned to each guideline on the same 7-point scale. Any major differences in the scores of the AGREE II items were resolved by a discussion between the appraisers.

#### Calculation of domain scores

The AGREE II tool consists of 23 key categories categorized into six domains: Scope and Purpose (item 1 through 3), Stakeholder Involvement (item 4 through 6), Rigor of Development (item 7 through 14), Clarity of Presentation (items 15 through 17), Applicability (items 18 to 21), and Editorial Independence (items 22 and 23). In addition to two global rating items, there are also two global rating items. The guideline's quality is addressed in each section. The two global items will be answered by confirmed appraisers once the 23 items are completed. As part of the overall appraisal process, appraisers should consider the standards used in the appraisal process when judging the overall quality of a clinical guideline. It is also important to note if they recommend the product based on its clinical performance. A score of 1 (strongly disagree) to 7 (strongly agree) is given to each item in this tool. Each of the 6 domains has a separate score based on the report's accuracy and quality. As well as standardizing the accepted scores for each domain and computing the overall mean scores for every domain, the results were analyzed.

McMaster University's Capacity Enhancement Program created AGREE II score concordance calculator to select scaled scores for each domain (22, 46). Based on the item scores for each domain, the quality score was calculated as a standardized percentage of the possible maximum:

Scaled domain score = (Obtained score – Minimum possible score) × 100 / Maximum possible score – Minimum possible score.

Maximum possible score = 7 (strongly agree) × 23 items × (3 or 5) appraisers.

Minimum possible score = 1 (strongly disagree) × 23 items × (3 or 5) appraisers.

Moreover, the domain scores should not be combined. During the final evaluation, the appraisers' final recommendation on how the guidelines should be implemented clinically is also presented (22).

#### Interpretation of domain scores

Appraisers' agreement between themselves was evaluated using the intraclass correlation coefficient (ICC) and was described as slight (0.20 ≥), fair (0.21–0.40), moderate (0.41–0.60), strong (0.61–0.80), or almost perfect agreement (0.81–1) (47). Previously, each clinical guideline's overall quality was determined by applying a 60% threshold to each domain's final score (22, 46). A guideline with a score of more than 60% across 5 or more domains is high quality, a guideline with a score of more than 60% across 3 or 4 domains is moderate quality, and a guideline with a score of <60% is poor quality. In addition, the overall quality has been measured as (mean ± standard deviation). Recommendations for the clinical performance of the guidelines have been expressed as suggested, recommend with changes, and not recommended. According to the previous articles, the scores of the domains were categorized as good (>80%), acceptable (60–79%), low (40–59%), and very low (<40%) (48, 49). The AGREE II instrument is widely used to evaluate the quality measure of clinical guidelines to evaluate methodological rigor and clarity of the guideline development process. This tool has been validated and tested for high trustworthiness with the exact framework to evaluate the quality of guidelines in six standardized domains while also providing a methodological approach for guideline development and scope (22). Terrace validated this instrument in a study as an international appraisal tool for estimating the quality of clinical guidelines in which 95% of appraisers considered the instrument valid for the appraisal of clinical guidelines. Moreover, the trustworthiness of its recognized components was acceptable with a score of 64–88% (22). In Iran, Rashidian and Yousefi-Nooraie (50) translated the AGREE tool into Persian, and its validity was confirmed by the cooperation committee of the Tehran University of Medical Sciences and the Ministry of Health and Medical Education. They translated the instrument independently and assessed face validity and fluency extensively during the translation process. A total of 11 guidelines were appraised across three specialties using the instruments. In the second appraisal, the raters discussed each guideline in groups and revised their scores individually. A total of 96 appraisals were conducted. In each time point, ICC (1,1) was calculated for domain scores received by a pair of versions. In addition, the reliability of the Persian version of the instrument and its English version was not found to be significantly varied after being compared with each other (51).

## Results

### Search results

The initial electronic database search produced 309 guidelines based on PRISMA (Preferred Reporting Items for Systematic Reviews and Meta-Analyses), and also the titles of all the clinical guidelines of the WHO were scanned (Figure 1). After the removal of duplicates and two rounds of article screening at the title (*n* = 94) and abstract (*n* = 53), there were 40 full texts reviewed. The steps to exclude the guidelines are fully mentioned in Figure 1.

### Definition of included guidelines

From the systematic literature search, we identified 40 guidelines. Several unrelated guidelines based on PIPOH (Population, Intervention, Professionals, Outcome, Health care setting) were removed during several meetings of the executive team, and 16 final clinical guidelines were sent to the evaluator from 2021/6/30 to 2021/8/7. Some of the guidelines were mostly applicable to patients diagnosed with severe and persistent mental disorders or common mental health problems (*n* = 5) or depression, violence, suicidal ideation, or attempt in patients with mental disorders (*n* = 4). Fewer guidelines were targeted to people diagnosed with schizophrenia or early psychosis (*n* = 3), BMD (*n* = 2), and directly about carers of patients (*n* = 2).

### Methodological quality of guidelines

As shown in Table 2, each guideline in this review received an AGREE II domain score. There were 7 high quality guidelines, 6 low quality guidelines and 3 medium quality guidelines (High quality means: >60% in 5 fields or more, average quality means: >60% in 3 or 4 fields, and low quality: >60% in 5 fields or less). The lowest mean quality score was in stakeholder involvement (20/37%), while the highest scores were in clarity of scope and purpose (96/21%) and presentation (98/14%).

**Table 2:** AGREE II domain and overall quality scores of included guidelines.

**Number**	**The title of the clinical guideline**	**Scope and purpose %**	**Stakeholder involvement %**	**Rigor of development**	**Clarity of presentation %**	**Applicability %**	**Editorial independence %**	**Overall guideline assessment %**	**I would recommend this guideline for use**	**Classification level**	**Quality report**
1	NICE (30)	91/6	80/5	69/7	72/2	64/5	54/16	54/16	Yes, with changes (4 out of 4 appraisers)	“Highly recommended”	high
2	NICE (31)	40/2	40/2	30/20	31/19	31/21	56/25	45/83	No (3 out of 4)	“Not recommended”	low
									Yes, with changes (1 out of 4 appraisers)		
3	NICE (32)	96/29	90/74	83/33	98/14	83/33	86/11	88/88	Yes, with changes (2 out of 3 appraisers)	“Highly recommended”	high
4	Crawford et al. (33)	88/88	81/94	85/41	90/27	66/66	68/75	83/33	Yes (1 of 4 appraisers)	“Highly recommended”	high
									Yes, with changes (3 out of 4 appraisers)		
5	NICE (34)	40/27	52/77	39/58	51/38	37/5	64/58	58/33	No (3 appraisers out of 4) Yes with changes (1 appraisers out of 4)	“Not recommended”	low
6	NICE (35)	94/44	86/11	80/20	65/27	57/29	83/33	70/83	Yes, with changes (4 out of 4 appraisers)	“Highly recommended”	high
7	NICE (36)	33/33	20/37	24/52	38/88	25	38/88	22/22	Yes (3 out of 3 appraisers)	“Not recommended”	low
8	Thornicroft et al. (37)	61/11	71/92	61/80	50	45/83	61/11	55/55	No (3 out of 4 appraisers) Yes with changes (1 out of 4 appraisers)	“Not recommended”	moderate
9	MOH (38)	41/66	45/83	45/31	72/22	45/83	56/25	50	No (3 out of 4 appraisers)	“Not recommended”	low
									Yes, with changes (1 of 4 appraisers)		
10	Santa Mina et al. (39)	64/81	62/96	70/83	79/62	63/88	61/11	72/22	Yes (1 of 3 appraisers)	“Highly recommended”	high
									Yes, with changes (2 out of 3 appraisers)		
11	Couroupis et al. (40)	76/38	72/22	63/54	50	44/79	54/16	58/33	Yes, with changes (3 out of 4 appraisers)	“Highly recommended”	moderate
									No (1 out of 4 appraisers)		
12	NICE. (41)	91/66	81/94	64/06	75	46/87	66/66	70/83	Yes, with changes (3 out of 4 appraisers)	“Highly recommended”	high
									Yes (1 out of 4 appraisers)		
13	de la Cámara Izquierdo et al. (42)	50	48/61	57/29	65/27	68/75	68/75	51/16	Yes (3 out of 4 appraisers)	“Not recommended”	moderate
									Yes, with changes (1 of 4 appraisers)		
14	Addington et al. (43)	41/42	33/33	39/06	48/61	32/29	64/58	50	No (3 out of 4 appraisers) Yes with changes (1 out of 4 appraisers)	“Not recommended”	low
15	Keepers et al. (44)	70/37	61/11	76/38	79/62	68/05	72/22	77/77	Yes with changes (2 out of 3 appraisers) Yes (1 out of 3 appraisers)	“Highly recommended”	high
**Mean scores**	65/49	62/03	59/41	64/51	52/11	63/79	58/29			

The answer given by each appraisers to the sentence “I would recommend this guideline” is listed in this table and the overall quality of each guideline is also known.

#### Domain 1 (Scope and purpose)

In the domain of scope and purpose, the mean AGREE II score was 65/49%. Guidelines with lower scores were generally described unclearly, including the disease stage and treatment phase of the target population.

#### Domain 2 (Stakeholder involvement)

Based on the included guidelines, the average quality score for stakeholder involvement was 62/03%. A total of 9 guidelines in this domain received a score of 60 or higher out of 15 sets. A number of reasons contributed to the guidelines' lower performance in this domain, including the lack of professionals and relevant stakeholders from their research teams.

#### Domain 3 (Rigor of development)

Among the included guidelines, the section on rigor of development received a mean score of 59/41%. This domain was dominated by three guidelines by NICE (32), Crawford et al. (33) and NICE (35) (>80%). In several guidelines, systematic search methods were used and formulate recommendations, an external review of the recommendations was obtained, and updates to the reports were considered.

#### Domain 4 (Clarity of presentation)

According to AGREE II, the guidelines scored 64/51% in the clarity of presentation domain. A majority of the guidelines (*n* = 9, >60%) met the quality threshold in this domain. In general, item scores are lower when different options or conditions for management are not adequately presented.

#### Domain 5 (Applicability)

In the domain of applicability, the guidelines included received a mean quality score of 52/11%. A score of over 60% was achieved by six guidelines. This domain included AGREE II items that often received low scores, including reviewing resource implications and describing auditing or monitoring procedures.

#### Domain 6 (Editorial independence)

According to AGREE II, the guidelines scored 63/79% for editorial independence. Major funding sources and conflicts of interest were acknowledged in most articles. Few guidelines explicitly noted that the funding source did not influence the content of the guideline.

#### Overall guidelines assessment

Over half of the guidelines (*n* = 8, 60%,) satisfied the quality threshold with domain scores of 60% for the overall guideline review (Table 2). These guidelines had a mean overall quality score of 58/29%. Ten of these guidelines focused on patients with common and severe mental disorders (*n* = 4) (31, 37, 41, 42), schizophrenia and psychosis (*n* = 3) (40, 43, 44), depression (*n* = 1) (34) and BMD (*n* = 2) (30, 38). The remaining five guidelines focused on violence and aggression (*n* = 2) (35, 36) and topics ranged from suicide care and assessment (*n* = 1) (39) and caring of patients with mental disorder and supporting caregivers (*n* = 2) (32, 33).

## Discussion

This study presents the first critical appraisal of guidelines with recommendations related to FCCC for individuals with chronic mental illnesses like schizophrenia, MDD, or BMD to our knowledge. It was found that 16 sets of guidelines published between 2009 and 2021 included at least one specific FCCC recommendation aimed at individuals after diagnosis of chronic mental illness.

These results demonstrate the need for better-developed guidelines addressing FCCC in populations with chronic mental illness. In addition, most guidelines focused on mental illnesses such as symptom management rather than specifically family or collaboration. The majority of guidelines were relevant to patients at the beginning of the severe mental illness care continuum, which demonstrates the need for customized FCCC guidelines and how HCPs can involve informal caregivers in their care. This study highlights, however, the need for further research on FCCC's role in managing chronic mental illnesses such as schizophrenia, MDD, and BMD. Despite the large differences in quality between the guidelines, stakeholders identified a few guidelines of acceptable quality for targeting FCCC recommendations for chronic mental illness populations.

For overall quality assessment, six guidelines met the quality threshold with domain scores of >60%. Stakeholders can use these findings to develop guidelines for clinical and research practice that are well-developed and suggestive. These guidelines were strongest in terms of scope and purpose, as well as clarity of presentation. Applicability received lower quality scores. Based on our search, no research has been done that has examined clinical guidelines with recommendations related to FCCC of patients with chronic mental disorders using the AGREE II tool.

An evaluation of the Evidence-Based Mental Health and Psychosocial Support (MHPSS) Guidelines was conducted in 2022 by Hans te Brake and his colleagues using AGREE-HS, a quality assessment tool with five core quality items focusing on (1) topic; (2) participants; (3) methods; (4) recommendations; and (5) implementation (52, 53) while we used the version of AGREE II that was updated on December 2018 to evaluate the guidelines. Among most included guidelines, FCCC recommendations for HCPs need considerable improvement regarding their applicability and implementation in practice. There are knowledge gaps associated with identifying the involvement of informal caregivers in the care of individuals with Schizophrenia, BMD, and MDD when applying FCCC recommendations.

Guidelines with a score of >60% in five or more areas in the quality report present strategies for implementing recommendations, such as family engagement training programs for HCPs (30, 32, 33, 35, 39, 41, 44). Relevant information on personalized care, such as psychological interventions, carers in care planning, assessing physical health, medications, supported employment programs, preventing violence and aggression, practice and educational recommendations about assessment of adults at risk for suicidal ideation and behavior and care plan, psychosis, treatment across all phases, substance misuse and primary care, psycho-education, and community treatment was also provided in several guidelines.

To facilitate the application of these guidelines to clinical practice, additional research is needed to determine practical knowledge translation techniques. Quality scores >60% were found for scope and purposes, Stakeholder involvement, the rigor of development, clarity of presentation, and editorial independence. It was found that several guidelines were lacking adequate information on the literature search methods and article selection criteria, how the evidence was described and graded, sufficiently as the exact process for developing recommendations.

In our analysis, several guidelines did not report on external reviews. Individuals with a research or clinical expertise in the area or those who benefit from using relevant guidelines can evaluate these recommendations thoroughly, comprehensively, and unbiasedly. The process for updating evidence-based guidelines is another important consideration. For example Crawford et al., which is included in this review, published an update to the 2021 survivorship guidelines in 2011 (33). For target users to be aware of current evidence-based best practices, it is essential to examine and revise recommendations occasionally due to the quick emergence of further evidence in this evolving domain of research (54, 55).

A key strategy to improve the rigor of guideline development in research is to use reproducible and systematic literature searches, provide adequate details about how recommendations were developed and modified, receive feedback from external reviewers, and update the guidelines regularly. The guidelines reviewed in this review included a range of practices regarding stakeholder involvement. A wide range of professions and expertise areas were represented on most guideline development teams. Despite the majority of selected guidelines incorporating findings on the care of individuals with Schizophrenia, BMD, and MDD, few recommendations directly addressed the way in which families should be involved in the care process. For the guideline development for the target population, it is important to explore and incorporate the views and preferences of people with Schizophrenia, BMD, and MDD, their informal caregivers, and HCPs. Thus, recommendations can be confirmed as applicable to the target population. Stakeholders should be involved at least within the external review process of guideline development, despite the benefits of integrating them throughout the process.

There were extensive reports on funding sources among the members of previous guidelines on editorial independence. Although specific conflicts of interest are not always addressed adequately, they can have an impact on the development and content of guidelines. Family involvement in mental illness research may present fewer conflicts of interest than in other fields, but they should still be documented as part of classic practices in guideline development. The clarity of presentation for the recommendations was practical in considerable guidelines reviewed.

An especially significant example of guidelines for individuals with Schizophrenia, BMD, and MDD where the FCCC recommendations and other suitable knowledge were efficiently identifiable and given by the NICE (2020) (32). This aspect is important for caregivers, patients, and actually, HCPs who may not have enough knowledge, time, or skills to search *via* lengthy, complex records for relevant knowledgeAn area of strength in the guidelines included in this review was the detailed overview of the purpose and scope of the recommendations. Most guidelines specified their objectives and health questions, by determining the general population, intervention, and outcomes under study. This knowledge would be beneficial in providing the correct application of the recommendations according to particular patient characteristics.

## Limitations

Limitations of our review contain possible selection bias in inclusion criteria. Guidelines that did not have recommendations on exact FCCC parameters were excluded. For example, 25 guidelines about managing mental disorders suggested, but did not include precise recommendations about FCCC and were unrelated to the PIPOH model of the study were excluded as these did not meet the criteria for our definition of FCCC, and including these was beyond the scope of this study's purposes and search strategy. Furthermore, guidelines that were not published in the English language were excluded. Regardless, this review helps to identify acceptable quality records within a set of newly disseminated guidelines that contain FCCC recommendations for people with Schizophrenia, BMD, and MDD. Further limitations are connected with the use of the AGREE II tool for the critical appraisal of the guidelines contained in this review. The mid-scores of the 7-point scale on the AGREE II tool have not been well defined for each object. There are no exact recommendations on solving domain and overall quality scores or setting precise cut-off scores to distinguish quality guidelines. Although based on earlier guideline evaluations, the use of the 60% cut-off score is somewhat incidental (46, 48). However, this tool allows an exhaustive assessment of guidelines and should be consulted in the development of prospective recommendations. Eventually, these results emphasize the necessity for further research utilizing rugged methods and examining FCCC, particularly in other chronic mental illnesses.

## Conclusions

Most of the guidelines designed newly include FCCC recommendations for individuals diagnosed with mental illness. These guidelines involve schizophrenia, MDD, and BMD and cover different broad topics (e.g., medication, care, treatment, etc.). According to AGREE II criteria, the stability of existing guidelines contains the title of scope and purpose, as well as the clarity of presentation. Improvement is needed in the applicability of guidelines. Also, there are limitations in the primary study reporting the recommendations, and guidelines of sufficient quality exist to stakeholders on FCCC for the people with Schizophrenia, BMD, and MDD. HCPs and informal caregivers can play an important function in helping these patients throughout the illness continuum and help from permit to well-developed and suitable care to solve research understanding on effective rehabilitation strategies, including FCCC.

## Suggestions for future studies

– Designing a family-centered collaborative care model for patients with chronic mental disorders.
– Development of clinical practice guidelines for family-centered collaborative care of other patients with chronic mental disorders.
– Designing a continuous training program for health care providers with a family-centered collaborative care approach.
– Designing a family-centered cooperative care training program for patients with chronic mental disorders.
– Implementation and evaluation of the impact of the application of the family-centered collaborative care clinical practice guideline for patients with chronic mental disorders referring to treatment centers.

## Data availability statement

The original contributions presented in the study are included in the article/supplementary material, further inquiries can be directed to the corresponding author/s.

## Author contributions

Conceptualization and data collection: RD and MF-M. Data analysis: RD, MS, and EM-S. Funding acquisition: MF-M. Methodology and writing—review and editing: RD, MF-M, and MS. Project administration and supervision: MF-M and MS. Validation: EM-S, MS, and MF-M. Writing—original draft: RD. All authors contributed to the article and approved the submitted version.
